# Microbes as vital additives for solid waste composting

**DOI:** 10.1016/j.heliyon.2020.e03343

**Published:** 2020-02-19

**Authors:** Mansi Rastogi, Meenakshi Nandal, Babita Khosla

**Affiliations:** Department of Environmental Sciences, Maharshi Dayanand University, Rohtak, India

**Keywords:** Environmental science, Environmental health, Environmental management, Environmental pollution, Environmental risk assessment, Environmental toxicology, Municipal solid waste, Food waste, Effective microbes, Additives, Composting

## Abstract

Composting is a natural process that stems through microbial succession, marking the degradation and stabilization of organic matter present in waste. The use of microbial additives during composting is considered highly efficient, likely to enhance the production of different enzymes resulting in better rate of waste degradation. In lesser developed countries, composting has emerged as a vital technology to recycle the biodegradable waste while generating a useful product. Depending on the composition of the waste material, it can either directly undergo composting or homogenized prior to secondary waste treatment methods such as landfilling. However, a relatively expensive downstream handling all along is a main hurdle towards economics of the process. Although basic methodology and recent approaches are known in crucial aspects of the process through various reviews, exploring the behavior of effective microbial additives will be resourceful. In this review, to fill in the gap, studies related to microbial composting of municipal solid and food waste were acknowledged. Here in, factors that could slow down the composting process and affect the compost quality were addressed. Lastly, the review pictured a positive simulation and stated how excellent results, can be achieved by microbial additives during composting.

## 1 Introduction

The MSW (Municipal solid waste) is a serious problem, emerging at an alarming rate in the megacities of the world as a consequence of overpopulation, urbanization, industrialization and the indiscriminate disposal of waste. In today's world, solid waste generated by the rapidly surging population in the megacities is of very high magnitude, mandating the practicing of solid waste management (SWM) strategies including; collection, transportation, processing and disposal of the solid waste (Raju et al., 2018). The municipal solid waste generally includes household and commercial refuse, consisting of degradable (paper, food waste, straw and yard waste), partially degradable (wood, and sludge) and non-degradable materials (leather, plastics, metals, glass, electronics) (Jha et al., 2003). Among these, the degradable wastes that constitute the major fraction of MSW load in developing countries, typically characterized by high water content (>60%) requires greater operating cost and lesser chances of material recovery (Wei et al., 2017).

The Ministry of Environment has released the solid waste management rules in 2000, for effective collection and disposal of municipal solid waste in India. Although, the existing solid management system has emerged successful in the last decade, still the system could be tailored as per the variable characteristics of urban waste. The modern solid waste management practices advocate material recycling, reduction, stabilization of solid waste prior to landfill disposal and energy recovery (Ionescu et al., 2015; Bong et al., 2017). However, these practices require proper certifications from government and may differ in developing and developed nations, rural and urban areas, residential and industrial setup.

## 2 MSW scenario in India

In recent decade, the rapid increase in human population and accelerated economy has caused an exponential increase in the waste generation rate. Approximately 1,88,500 tonnes (68.8 million tonnes per year) of municipal solid waste is generated per day in urban India (Gupta and Arora, 2016). However, only 24% of this humongous waste is processed, treated and disposed off by suitable methods. The waste disposal in India is mainly done by open dumping, landfilling, composting and incineration; open disposal being the cheapest and most common method currently practiced (Ghosh et al., 2015). After open dumping, waste landfilling is majorly undertaken for waste treatment and disposal, but requirement of larger areas limits the disposal of MSW especially in bigger cities (Mani and Singh, 2016). It is anticipated that by 2047, 1,400 sq.km of land area would be required for landfilling of MSW generated in India and this accounts for almost the combined area of three most populous Indian cities (Annepu, 2012). Further, landfills produce high levels of toxic secondary pollutants such as odors, leachate and greenhouse gases that restricts its use for waste treatment. This state of affairs recommends an applied process, such as MSW pretreatment done to homogenize the waste and ease the waste treatment process through certain biological technologies, before landfilling (Białowiec, 2011; Dziedzic et al., 2015). In spite of all the information and resources, lack of technical expertise creates a lot of pressure on municipalities and local government, to find a sustainable and cost-effective waste treatment method (Kaushal et al., 2012).

**Figure 1:**
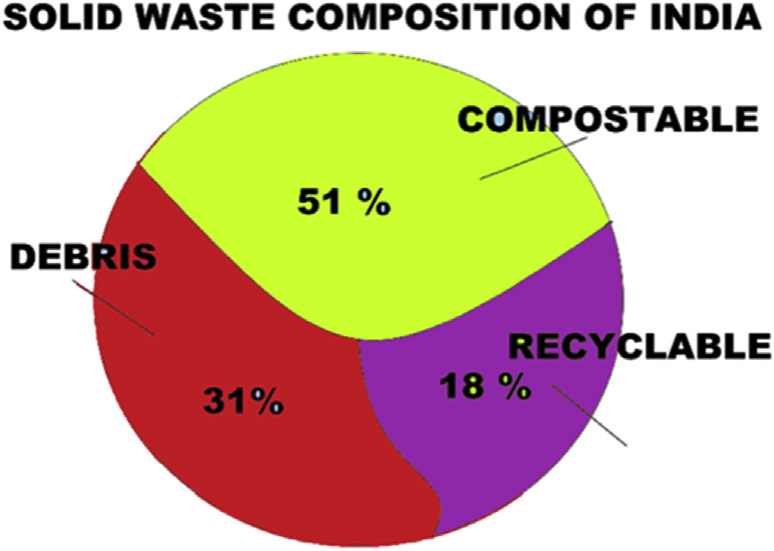
Solid waste composition of urban India.

Among all the MSW recycling methods in context to organic matter, Wei et al. (2014) and Storino et al. (2016), spelled out composting as the most preferred, ecofriendly and economically viable waste treatment technology when managed effectively. Composting is the most extensive applicable process to manage these wastes particularly in case of Indian genera, where 50–60% of MSW (C/N ratio 23) collected is biodegradable (Figure 1). In addition, switching the organic raw material from landfills to composting has several environmental benefits as well. Besides reduced landfill greenhouse gases (GHGs) emissions, there is improvement in soil properties like texture, porosity, organic matter and available NPK content of soil for agricultural applications (Kjerstadius et al., 2016). Conclusively, we need to understand the know-how for this natural, wet waste recycling process and implications of the operative modifications to upgrade the waste recycling system.

## 3 Anatomy of composting process

Composting can be defined as an aerobic, biochemical and microbial process that implicates the hydrolysis of organic fraction into stable and sanitized residue; humus (Wei et al., 2017).

Here in, microbes carry out the decomposition of organic matter by utilizing carbon and nitrogen as the energy sources along with oxygen and water, ensuring the production of water, carbon dioxide, heat, and soil-enriching compost. The derived compost possesses a significant concentration of biologically stable humic substances, acting as excellent soil amendment (Białobrzewski et al., 2015). During the process a spontaneous rise in temperature, helps to eliminate the pathogens, making the generated compost safer for use.

The process of composting includes majorly three phases (i) an initial mesophilic phase, where degradation of simple compounds like sugars, amino acids, etc. is carried out by mesophilic bacteria and fungi rapidly elevating the temperature; (ii) Second phase is thermophilic phase, where thermophilic microbes degrade the organic matter (fats, cellulose, hemicellulose and lignin). During this phase, organic carbon content is decreased in the feedstock ascribed to the metabolic activities of heat-tolerant microbes. Lastly, (iii) cooling phase is characterized by a subsided microbial activity and decreased temperature. Within this, the compost mass is recolonized by mesophilic micro-organisms that degrade the residual sugars, cellulose and hemicellulose, materializing humic-like substances (Albrecht et al., 2010). This is followed by a declined rate of organic matter degradation and an increased rate of humification and polymerization of the organic compounds.

During composting, the microorganism's succession is the key for an effective management of the process. The appearances of certain microorganisms influence the rate of biodegradation and compost maturity, reflected by the quality of the generated compost (Jurado et al., 2014). Furthermore, microbial inoculants influence the process of composting by altering the cellulose, hemicellulose and lignocellulose breakdown process, causing alterations to the temperature and nitrogen levels throughout the composting process. Even though, composting is supposed to be an oxygen-demanding (aerobic) process (Pepe et al., 2013), anaerobic organism like *Clostridium* sp. has been alluded in the process as well (Franke-Whittle et al., 2014; Bhatia et al., 2013).

### 3.1 Factors affecting the composting process

**Figure 2:**
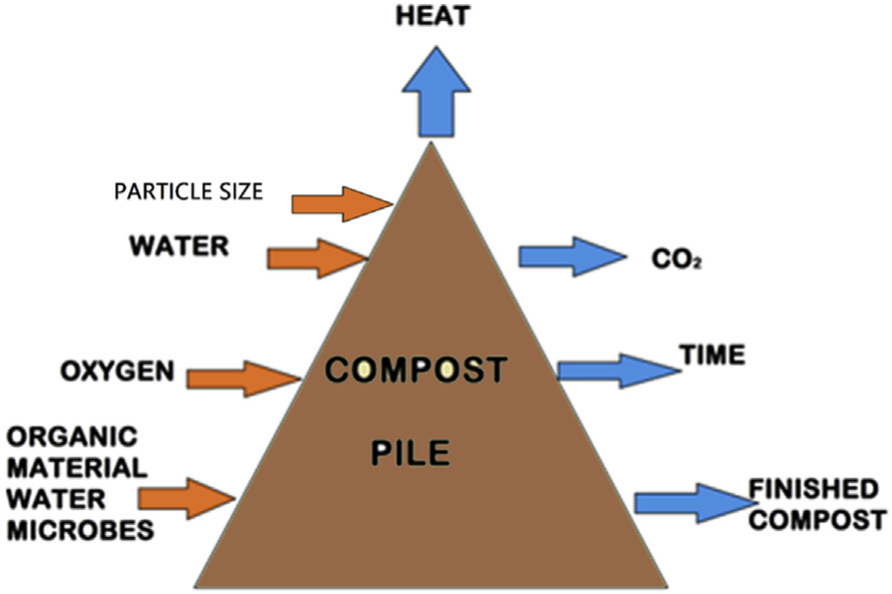
Components of composting process.

In the broadest terms, composting is affected by the factors, classified into two groups (i) depending on the formulation of the composting mix, such as nutrient balance, pH, particle size, porosity and moisture; and (ii) depending on process management, such as O_2_ concentration, temperature, water content and compaction (Li et al., 2013). Perhaps, the control on parameters such as pH, bulk density, temperature, porosity, nutrient content, C/N ratio, particle size, moisture and oxygen supply are crucial to get an exact idea about the desired optimal process conditions (Figure 2). Within composting, microbes required C, N, P and K as major nutrients (degradable organic-C) for energy supplement and developmental activity (Iqbal et al., 2015). Besides stated factors, Bernal et al. (2009), suggested that waste degradability throughout composting may also vary depending on the chemical constituents of the waste, natural load and microbial efficiency in the compost matrix. Likewise, environmental conditions directly influenced the microbial activity and the rate of organic matter degradation during composting (Hueso et al., 2012). Prevailing weather conditions (temperature and humidity) of the study area may also be relevant for the same.

a) C/N ratio

A nutritional balance in form of an optimum C/N ratio is essential to formulate an efficient compost mix. As composting proceeds with time, variations in C/N projected the rate of organic degradation as governed by the extent of carbon transformed to CO_2_. Ideally, necessary C/N ratio lies in the range, 25–35; stating that micro-organisms required 30 parts of C per unit part of N (Kutsanedzie et al., 2015). Nevertheless, some authors observed good results even with an initial C/N values between 20–50 (Kumar et al., 2010; Yang et al., 2015; Petric et al. (2015)). A higher C/N ratio (compared to recommended) slowed down composting speed and reported nutrient deficiency to microbiota, due to excessive accumulation of the substrates. While, a lower C/N resulted in increased N content per C (degradable) and inorganic nitrogen, likely to be lost as ammonia through volatilization or leaching (Kutsanedzie et al., 2015; Zhang et al., 2016). Subsequently various undesirable compounds (such as odors and salts) unfavorable for plant development are released (Mohee et al., 2015; Onwosi et al., 2017). A general trend was observed with declined C/N ratio throughout the process attributed to a higher waste degradation (carbon) to mineralization (nitrogen) ratio (Yang et al., 2015; Wang et al., 2016; Rastogi et al., 2019a). Hence, to optimize C/N within composting, wide variety of bulking agents are recommended as additives to waste (e.g. sawdust, rice husk, peanut shells and wood chip). They are known to develop enhanced porosity in the feedstock material and homogenize the waste before composting (Wang et al., 2015; Zhang and Sun, 2016).

b) pH

Articulating the contributing factors for composting, pH is not pondered vital (initial stage) with most of the raw materials already assorted within the recommended pH range (Rich and Bharti, 2015). Somehow, a decreased pH amidst composting can be related to the volatilization (ammonia) and microbial nitrification producing larger amount of CO_2_ and acids (Wang et al., 2016). While, protein mineralization (production of ammonia) and the impeded N, lost through ammonia volatilization (Guo et al., 2012) at subsequent stages of composting explains the elevated pH (>8). For composting, ideal values for pH generally range from 5.5-8.0 (Zhang and Sun, 2016a, b); but Bernal et al. (2009), opined that a pH value between 6.7-9.0 is effectual to promote good microbial action. Contrarywise, Zhang and Sun (2016a, b) observed reduced microbial activities and an exceeded pH (9.0), manifested by the presence of nitrogenous compounds in the compost mass. The elevated pH causes alkalization of compost mass that may hinder the survival of pH sensitive microorganisms, profusely contributing to sanitation of the compost (Hachicha et al., 2009). In addition, pH and temperature can collectively affect the waste degradation process, evidenced by the co-existence of numerous microbial communities at different pH and treatment setups (Sundberg et al., 2013).

c) Moisture content

The moisture conditions essentially impinge microbial activity, oxygen uptake rate, temperature and the porosity level within composting (Petric et al., 2015). An effective composting will need about 50–60% (v/w) moisture content in accordance with the composition of the raw material (Bernal et al., 2009). Unlike pH, an inverse relation exists between moisture content and temperature, exhibiting an increase in temperature as the moisture content drops (Varma and Kalamdhad, 2015). The elevated temperature resulted in higher evaporation, causing a drop-in the rate of organic matter decomposition. Thus, rewetting of treatment piles must be done to maintain adequate moisture conditions and for proper functioning of the waste microbiota. Conversely, moisture content higher than required during composting could generate water logs with prevalent anaerobic conditions that might halt the procedure (Makan et al., 2013). As an exception, lignocellulosic composting with raw material such as rice straw, a higher moisture content is desired to soften the strong fibrous material, implicating a positive effect on the process (Kádár et al., 2012).

d) Aeration/O_2_ supply

Aeration supplemented through proper O_2_ supply is another critical aspect, provides oxygen mainly for micro-biological processes, temperature control, moisture optimization and removal of excessive carbon dioxide. Latifah et al. (2015), proposed that oxygen concentration should range between 15-20 % for a desirable composting. Here in, the oxygen concentration is directly correlated to the microbial dynamics (Nakasaki and Hirai, 2017) and temperature (maintained below 60–65 °C), to ensure enough oxygen is supplied within the process (Latifah et al., 2015). A sufficient aeration at an early composting stage, shortened the process time (waste to stabilize) resulting in complete transformation of carbon (C) to carbon dioxide (CO_2_) and reduced methane emissions. Whilst, excessive aeration within the matrix could result in faulty composting, causing drastic effects on the waste decomposition rate (Awasthi et al., 2014). In a study, a higher aeration rate (0.2–0.6 L min^−1^ kg^−1^ OM) during waste composting moderated the C/N ratio, NH_3_ generation and odour release, but adversely affected the maturity of compost (Zhang and Sun, 2016). Whilst, composting at a lower aeration rate (<0.2 L min/kg/OM), resulted in slower organics degradation, reduced NH_3_ production with a significant decline in temperature, moisture and heat loss, ultimately influencing the microbial population (Guo et al., 2012). To sustain optimum aeration and achieve better stabilization and sanitization of waste, a turning regime must be maintained for the compost mass. Furtherance, a strong link between turning frequency and few physicochemical variables could be used to indicate the compost maturity (Getahun et al., 2012). For instance, turning frequency affects; pH, total nitrogen content, moisture content, C/N ratio, dry matter, total carbon, and temperature within the composting pile. A weekly turning regime for waste was reported to be effective for a faster organics' degradation (Mohee et al., 2015). A comparative MSW composting study involving, two turning regimes for bacterial succession observed a three-day per week turning regime of most significance, while daily turning did not fit well for the bacterial succession (Awasthi et al., 2014). Relatively, mixing the composting mass for 30min daily improved the co-composting of MSW and poultry manure (Petric et al., 2015). In addition, forced aeration and pile turning together have shown positive results on FW composting, depicted in final compost quality (Li et al., 2015; Rasapoor et al., 2016).

e) Temperature

Akin moisture content and aeration, temperature as well stimulates the growth and metabolic activity of the microbial community within compost mass. It can directly affect the biodegradation rate of the organic matter during composting (Waszkielis et al., 2013). Therefore, a need for temperature regulation is suggestive to control the process pace. The ambient temperature hastened the degradation of organic substrates and increased their biodegradability rates during MSW composting (Rastogi et al., 2019b).

Salama et al. (2017), pointed out, temperature ranging between 50- 55 °C favored waste decomposition and ensured maximal sanitization during composting. Furthermore, temperatures and process time collectively work for complete elimination of the pathogens in the compost mass (Pandey et al., 2016). Conversely, excessive heat (i.e. greater than 70 °C) for a longer time can deactivate the micro-organisms (fungi, actinomycetes, and bacteria) during composting, necessitating the maintenance of temperature regime (Varma and Kalamdhad, 2015). The excessive heat can be removed by regulating the size and shape of the composting mass through turning operations, resulting in improved cooling and temperature redistribution (Chowdhury et al., 2013). MSW composting studied by Troy et al. (2012), reported a quick rise in the temperature (reached 50 °C on day 8) and heat generation due to rapid microbial degradation of sugars, proteins and fats. In a similar study, Jara-Samaniego et al. (2017), performed composting on the MSW generated in the Chimborazo Region (Ecuador) with six piles (Piles 1, 2 and 3 with municipal solid wastes) from the Riobamba landfill and Piles 4, 5 and 6 developed by source-separated wastes. Results conversed, highest thermal peak (66 °C) by Pile 4 (FW), probably associated with higher sugar content and water-soluble carbon.

f) Particle size

Particle size in compost mass ensures the porosity level, to ensure suitable aeration and regulate the gas/water exchange (Zhang and Sun, 2016). An appropriate particle size and shape is an important factor to estimate operational costs of the process (Wang and Ai, 2016). Ge et al. (2015), pointed out, ‘sieving’ as a fundamental method to determine optimum distribution of the particle size in a compost mass. An appropriate particle size can be achieved by shredding and chipping of the waste into smaller pieces. This ensures a more available surface area for better microbial activity during composting, resulting in speedy degradation. A small particle size (compared to normal) instigated initial compaction of the feedstock, developing a subsequent risk for prevalence of anaerobic conditions later (due to clogging of the small air spaces with water). Whereas, larger particles processed a smaller surface area, being less accessible for microbial action and developed big air pockets decreasing the matrix temperature, resulting in slower decomposition of organic matter (Verma and Marschner, 2013). Finest degradation was achieved with waste particles sized at 25 mm, that offered suitable physical and chemical conditions for bioactivity during a tobacco composting process (Zhao et al., 2017a).

g) Bulking agents

During composting, modifications in the MSW properties are brought up by the added bulking agents. In this case, wood chips, sawdust, rice husk and cornstalks are common bulking supplements that demonstrated an efficient composting of waste (Yang et al., 2013; Zhang et al., 2013). However, large wood chip rather than wood shavings or sawdust, would reinforce good aeration through the compost pile and provide lesser carbon per unit mass. Awasthi et al. (2015), showed that co-composting of the organic fraction of municipal solid waste (OFMSW) with wood as bulking waste, generated a well matured compost within 28 days. Likewise, Silva et al. (2016) used carbon sources and wood chips as bulking agents and assessed the feasibility of co-composting on the quality of poultry manure compost. He pointed out an enhanced waste degradation process and the final compost quality met the recommended standard values.

## 4 Role of microbes/microbial dynamics during composting process

The dynamics or succession of a microbial community within composting, reflects their degradative capacity for the compost mix (Ling et al., 2014). Along the process, variations produced in a microbiome depend extremely on composition of the raw materials and nutrient supplements, environmental conditions (ambient or trial) and interactions among all these factors. Here in, Bacteria and fungi are the most abundant and fastest emerging microorganisms during composting. The substrates utilized and the microbiota involved within the process have a great influence on the quality of the formed compost (Villar et al., 2016). They promote organic degradation within composting by releasing various substrate based hydrolytic enzymes (Echeverria et al., 2012), that break the complicated structured molecules, forming water-soluble compounds (Lee, 2016). Besides metabolizing the organics, they produce simple usable compounds that enhance the agricultural possibilities and stabilize the natural ecosystem when added to soil.

The mapping of diverse physiological microbial profiles in composting can portray a good picture of compost maturity events. An initial profiling showed an expected decrease in the microbial biomass, associated with changes in C/N ratio and temperature of the composting mass (Karak et al., 2014). At mid mesophilic stage of composting, bacterial population continued to proliferate with production of more enzymes that resulted in proper humification (Vargas-García et al., 2010). Finally, microbial mass underwent a gradual decline at the cooling or maturation phase of waste composting. Generally, the fungal abundance observed throughout the process was lower compared to the bacterial population (Chandna et al., 2013). Certain modifications in the process integrating, bulking agents as addons to the substrate (such as rice husk, saw dust, wood chips and others) can develop an efficient microbiota. This would further optimise the C/N ratio and retain quality of a compost (Zhang et al., 2013; Anwar et al., 2015; Yuan et al., 2015).

## 5 Microbial additives

**Figure 3:**
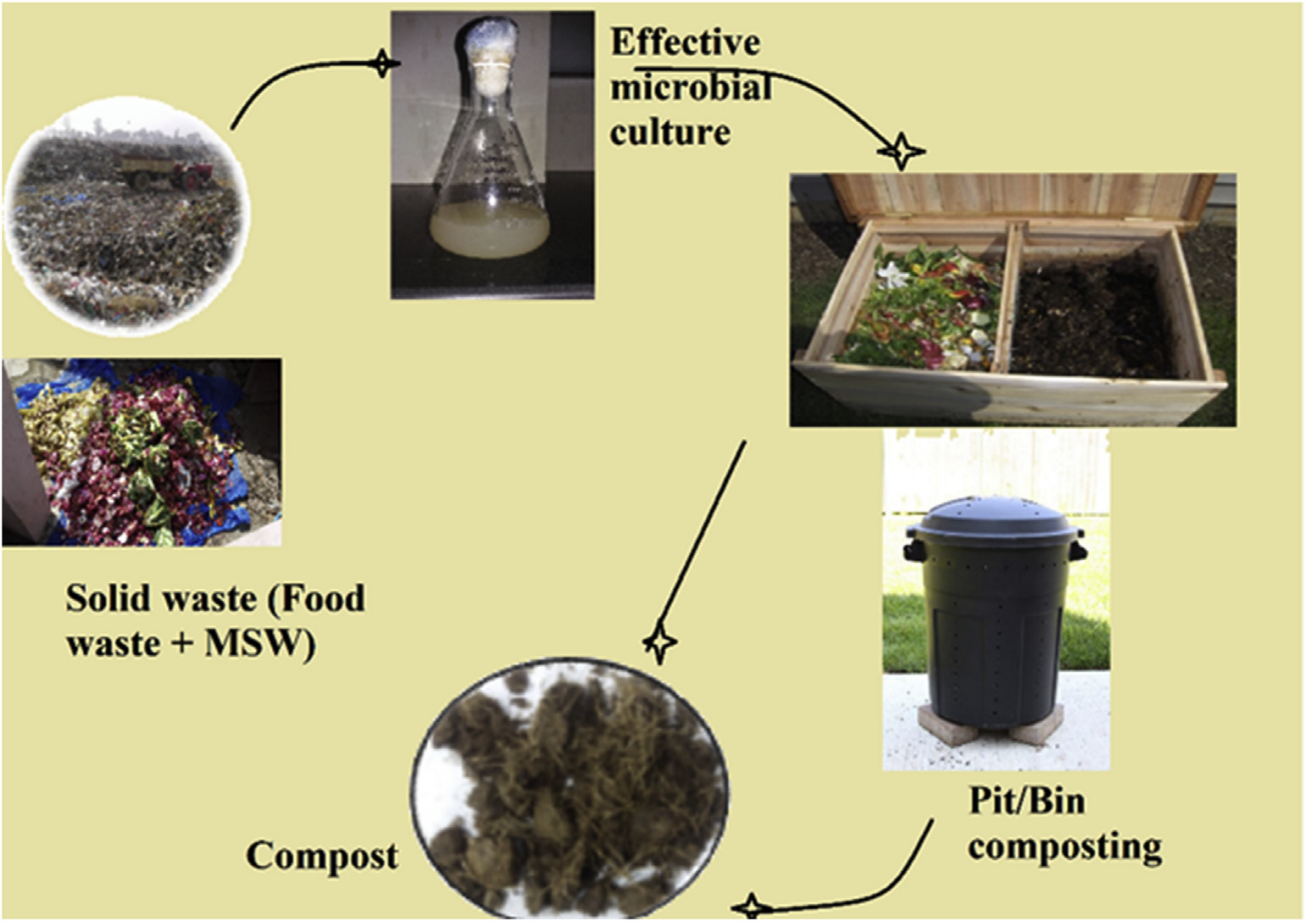
EM aided MSW Composting Operation.

Composting of the organic waste, occurs naturally by wet decomposition, but we lack a consensus deeming the efficiency of inoculation to this process. An EM culture is a mix of non-dominant and dominant microbes, where the former plays a more active role. A better clarification on this can be given by several studies (Karnchanawong and Nissaikla, 2014; Onwosi et al., 2017; Manu et al., 2017; Rastogi et al., 2019a, Rastogi et al., 2019b), that revealed how inoculation with efficient microbes (EM) additives, to the treatment mixtures enhanced the waste degradation rates. Further, these additives can either be isolated from the microbial communities according to specific degradative functions or developed through culture mixtures such as soil, cow dung, and straw etc. (Liu et al., 2011). To specify, the added inoculum might be a single strain of EM (Zhao et al., 2016; Hou et al., 2017; Nakasaki and Hirai, 2017), a commercial mixture of strains (Zhao et al., 2017; Rastogi et al., 2019a, Rastogi et al., 2019b) or a compost taken at maturity (Karnchanawong and Nissaikla, 2014; Kinet et al., 2015). It is known that major portions of the MSW (organic portion) is plant biomass, processed collectively by the synergy of cellulase enzymes. Yet, only a few microbial strains capable to secrete cellulase enzyme and degrade MSW through cellulose hydrolysis are in light (Gautam et al., 2012). Few known potent cellulose producing bacteria include; *Cellulomonas, Pseudomonas, Bacillus spp*. and *Thermoactionmycetes*. Likewise, fungal species *Aspergillus*, *Trichoderma*, *Sclerotium* and white-rot fungi, produce extracellular enzymes accountable for cellulose and lignin degradation during composting (Awasthi et al., 2015). Compost quality can be assessed by monitoring the changes in biological characteristics (microbial succession) occurring within the process. The compost can be verified in terms of pH, C: N ratio, color, electric conductivity (EC), humic substances (HS), pathogenic activity, germination index (GI) and total NPK contents (nitrogen, phosphorus and potassium). A generalized operational pattern for a microbial aided composting has been shown in Figure 3.

The microbial additive to a compost mix, affects the temperature profile and ammonia emissions owing to the proliferated mesophilic and thermophilic bacterial populations (Barthod et al., 2018). In addition, enhanced enzymatic activity and minimized initial lag time of the biological process, accounted for the accelerated composting by these effective microbes (Saad et al., 2013). It can also effectively reduce the odorous emissions, mainly volatile organic compounds (VOCs) and generate a compost with a higher nutritional value (Jusoh et al., 2013; Maulini-Duran et al., 2014). Few other significant sources of such emissions include ammonia (nitrogen-based odorous compound) and sulfur-based emissions (Zhang et al., 2013), once proteins get degraded during composting. A positive effect on emission of odorous compounds such as VOCs and ammonia, was detected during kitchen waste composting Shao et al. (2014) and Yuan et al. (2015). In addition, Charles and Ho (2017), opined that EM mixed to the organics, enhanced the degradation rate and reduced the odorous emissions as well. Another home scale composting of FW carried out to identify the role of EM in pacing the degradation process, achieved higher temperature, with suppressed foul odour and enhanced humification process for EM-Compost (Wang et al., 2011).

Certain composting investigations, revealed the influence of microbial inoculation on the compost quality as well. Usually, a significant reduction in the operation time (due to microbial action) of the degradation process, evidenced a good quality compost (Abdullah et al., 2013 and Zhao et al., 2016). A study by Wei et al. (2019), investigated lignocellulose degradation to assess the effect of actinomycetes inoculation on the substrate (FW). The results showed that actinomycetes inoculation accelerated production of the key enzymes, including CMCase, Xylanase, lignin peroxidase etc. and increased the rate of organic matter degradation. An actinomycetes inoculum (four cellulolytic strains) was inoculated into co-composting of dairy waste at different phases. The results revealed improved cellulase activities and an accelerated cellulose degradation, increased the content of humic substances (Zhao et al., 2017b). Manu et al. (2019), carried out decentralized composting of household wet biodegradable waste (HWBW) in the recycled plastic drums, inoculated with microbial inoculums. The added inoculum, reduced the composting time period to 30–36 days and produced a pathogen free compost. Awasthi et al. (2015), performed a co-composting experiment where feedstock material inoculated with mixed microbial culture (*Phanerochaete chrysosporium, Trichoderma viride* and *Pseudomonas aeruginosa*), was an effective tool to facilitate shortened composting period. Co-composting of MSW with sludge and mixed microbial culture (*Bacillus casei, Candida rugopelliculosa, Lactobacillus buchneri, Trichoderma* and *white-rot fungi*) drastically reduced the nitrogen loss and enhance the mineralization rate (Awasthi et al., 2016). Following the same approach Varma and Kalamdhad (2015), studied carbon decomposition during drum composting of waste with a fungal additive, *Phanerochaete chrysosporium* (white-rot fungus), where inoculation increased the waste decomposition rate within a shorter span.

An analogous study assessed the influence of microbial inoculation to OFMSW and assessed the changes in C/N thru composting. Setup for the investigation consisted of three lab scale aerated bioreactors (A-inoculated with the *Aspergillus niger*, B- inoculated with old compost and C- control). Results showed a decreased C/N (63.37%, 59.6% and 46%) for bioreactors B, A and control, respectively with a maximum temperature (59 °C) in reactor B (Heidarzadeh et al., 2019). Furthermore, a reduction in process time (18 days) concluded that microbe aided composting is an economic viable technique. An MSW study was done by Martínez Valdez et al. (2015), to check the effect of pH, C/N ratio, temperature and microbial consortium on organic fraction mineralization (CO2 production and formation). It was found that highest CO2 rate (5.28 d/1) was produced at C/N ratio 30, 27 °C temperature and 8% inoculum addition. While, the highest hydrolytic enzyme production was at 50 °C, established from the amount of reduced sugar. Manu et al. (2017), performed a modified drum composting to check the additive effect of improved natural air circulation and microbial inoculation on FW. The inoculation of microbes resulted in an early thermophilic phase, positive self-heating test, higher germination index (>80%) and produced a mature compost after 60 days. In another FW composting study, microorganisms when inoculated to waste for recycling and valorization, enriched richness and diversity of the microbial community, decreased C/N ratio, organic matter and cellulose content (Wang et al., 2019).

Co-composting with different microbial combinations at distinct stages might have contrasting effects, depending on the substrates. Voběrková et al. (2017), inoculated MSW with consortium of white-rot fungi *Fomes fomentarius*, *Phanerochaete chrysosporium* and *Trametes versicolor* in different combinations, achieving best results for mixed consortia composting. A lignocellulosic waste composting study determined the effect of *Trametes trogii* (white rot fungus), during different composting stages on the lignin and cellulose decomposition. Inoculations done at 0, 120 and 180 days showed that, lignin degradation paced maximum at the maturation phase for all the treatments (Fersi et al., 2019). Xi et al. (2012), studied the effect of initial-stage, two-stage, and multi-stage inoculations on the composting efficiency of MSW, where process efficiency was maximum for multi-stage microbial inoculations. A multi-stage inoculation improved the microbial diversity indexes, avoided the competition between inoculations and indigenous microbes, and enhanced the process efficiency (Xi et al., 2015).

The efficacy of EM addition to different solid waste and changes on the parameters was summarized and categorized in Table 1.

**Table 1:** Summary of the studies by different authors for various Solid waste as compost feedstocks.

Compost feedstock	EM Description	Impact on the overall composting process	References
Municipal solid waste (MSW)	Cellulolytic microbial inoculum (Phanerochaete chrysosporium and Trichoderma reesei)	Rapid composting as indicated by the reduction (below 20) in C/N ratio	Raut et al. (2008)
Food waste	Thermo-tolerant lipolytic actinomycete, Thermoactinomyces vulgaris A31	TOC, C/N ratio, CO_2_ evolution, and enzymatic activities (dehydrogenase, polyphenol oxidase, urease) decreased, pH, total nitrogen content, germination rate, and germination index increased.	Guangrueike et al. (2010)
Wheat straw	A cellulolytic consortium of Trichoderma sp., P. Chrysosporium and A. Oryzae	Enhanced enzyme production and synergism of enzymes and early maturity of compost	Hui Lin et al. (2011)
(Common organic wastes), fruit wastes, vegetable wastes, leaves, hay, newspaper, wheat straw and rice husks,	Bacillus subtilis and Pseudomonas	Reduction in C/N ratio, NH_4_^+^ and NO_3_^−^ ion concentrations and increased compost maturity	Pan et al. (2012)
Municipal solid waste	Mixed culture (*Nitrobacter* and Thiobacillus, lignin decomposition composite and fungi)	Improved humification degree of the composting products and increased efficiency of composting process	Xi et al. (2012)
Food waste	Yeast strain *Pichia kudriavzevii*	Increase in pH, temperature and accelerated the composting process	Nakasaki et al. (2013)
Agricultural waste composting	Cellulolytic And Deodorising Bacteria (P. Chrysosporium	Increasing pile temperature, enhancing the substrate utilizability, and changing other physico-chemical factors.	Chen et al. (2013)
Organic fraction of municipal solid waste (OFMSW)	Trichoderma viride, Aspergillus niger and Aspergillus flavus	Temperature, pH, TOC, TKN, C/N ratio and germination index, high degradation of organic matter and early maturity	Awasthi et al. (2014)
Kitchen-waste	Bacillus thermoamylovorans, Mixed Bacillus species (such as B. Brevis, B. Coagulans and B. Licheniformis	Composting process efficiency increased	Abdullah et al. (2013)
Food scraps and dry leaves	Lactic acid bacteria, photosynthetic bacteria and yeast	The C/N ratios of composts stabilized early with highest volatile solid mass reduction indicating mature compost.	Karnchanawong and Nissaikla (2014)
Food waste	Lactic acid bacterium *Pediococcus acidilactici*	Enhanced the proliferation of fungi having the ability to degrade organic acids and organic matter degradation in the composting was accelerated.	Nakasaki et al. (2015)
Msw	Cellulolytic consortium of Clostridia	Improved anaerobic digestion of cellulosic biomass	Kinet et al. (2015)
Organic Fraction of Municipal Solid Waste (OFMSW)	Cellulolytic EM	Rapid mineralization, increased CO_2_ production rate, stabilized C/N ratio and increased release of reducing sugars	Martínez-Valdez et al. (2015)
Food waste (FW)	Ligno-cellulolytic Consortium	Lowered extractable-Na (ext-Na) and electrical conductivity (EC) indicating compost maturity	Xu et al. (2019)
Agricultural waste composting	Phanerochaete chrysosporium	Reduction in C/N ratio, total organic matter, temperature and soluble-exchangeable Pb	Huang et al. (2015)
Empty fruit bunches	*Trichoderma*	C:N ratio stabilized, increased nitrogen (N), phosphorus (P), and potassium (K) were found in compost, enhanced soil micronutrient, plant growth performance, and crop yield production	Siddiquee et al. (2016)
Organic waste	Actinobacteria agent including *Streptomyces* sp. and *Micromonospora* sp	Improved the actinobacteria community diversity particularly in the cooling stage of composting and accelerated degradation of organic matters (OM) especially celluloses.	Zhao et al. (2016)
Wheat bran	*Bacillus subtilis* and *Chaetomium thermophilum*	Accelerated the degradation of proteinaceous compounds and the formation of complicated humic-like materials, high composting efficiency and degree of humification	Wang et al. (2016)
Organic fraction of municipal solid waste	White-rot fungi (*Phanerochaete chrysosporium, Trametes versicolor* and *Fomes fomentarius*),	Accelerated degradation of solid waste as indicated by changes in C/N, electrical conductivity and ph. Higher degrading ratio and a better degree of maturity, increased enzymatic activities (especially dehydrogenase and protease) and a suitable germination index	Voběrková e*t al*. (2017)
Lignocellulosic waste (LW) and the organic portion of municipal solid waste (OPMSW)	MI (cellulolytic and lignocellulolytic)	Significant positive effect on the composting of LW Compost quality parameters stabilized: pH, germination index, nitrogen content, phosphorus content, potassium content, C/N ratio; composting parameters: temperature, odour, enzymatic activities, organic matter content, microbial population, volume reduction, humification,	Fan et al. (2017)
Organic waste	Cellulolytic thermophilic actinomycetes	Increased content of humic substances and alleviated CO_2_ emission during composting.	Zhao et al. (2017)
Food waste	Lactic acid bacteria, yeast and phototrophic bacteria	Microbial population, humic substances, biological parameters (lignin, cellulose and hemicellulose) and germination index showed non- phytotoxic and matured compost	Manu et al. (2017)
Municipal sludge and solid waste	Microbial inoculums originated from sludge and MSW	Increased enzyme activity, composting stability and maturity (C:N ratio, and germination index)	Li et al. (2017)
Municipal solid waste	Psychrotrophic bacteria	Temperature, moisture content, pH, electrical conductivity, C/N, ammonium nitrogen, and nitrate nitrogen indicated that the compost had reached maturity and enhanced the stability of the microbial community structure.	Hou et al. (2017)
Food waste	Mesophilic yeast *Pichia kudriavzevii*	Promoted the degradation of organic matter and accelerating the composting process	Nakasaki et al. (2017)
Kitchen waste	EM	Higher temperature at the early stage, a greater fat reduction with foul odour suppressed, enhanced humification process and.	Van Fan et al., 2018a, Van Fan et al., 2018b
Municipal solid waste compost	*Aspergillus Niger*	C/N decreased and reduction in process time	Heidarzadeh et al. (2019)

### 5.1 Cellulolytic and lignocellulolytic process

In composting, the major befalling biological process is cellulolytic governed by the cellulase processing capacity of the bio-agents (Gupta et al., 2012). Here in, microorganisms are the bio-agents that degrade cellulose and lignin components within the waste matrix. These indigenous micro florae, likely generate higher enzymatic levels that ultimately pace up the composting process. However, only few microbes utilize cellulose efficiently, as a substrate differing in the type and activity of cellulases for degradation activity (Kumar et al., 2010). A study by Cao et al. (2013), reported that the inoculation of *Aspergillus fumigatus* F12 strain degraded cellulose, before other biopolymers.

Among cellulolytic microbes*, Bacilli* are the mesophilic bacterial species that degrade proteins, peptones, amino acids, and blood meal. Along with cellulose degrading microorganisms (fungi and bacteria) certain chemicals can augment the activity of released hydrolytic and/or oxidative enzymes, liable to depolymerize and transform the lignocellulolytic waste components to organic acids. Several studies indicated the correlation between microbial diversity and degradative capacity of lignocellulosic microbes, to generate humic substances (Jurado et al., 2015; Zhao et al., 2016; Wu et al., 2017; Villar et al., 2016). Considering the lignocellulosic fraction of the waste, several trial schemes have been executed via lignocellulosic microorganisms, for improved degradation rates (Manu et al., 2017, 2019; Zhao et al., 2016). Most of these studies reported positive outcomes and derived a high-quality final compost; however, not all the studies succeeded.

Consonant with the literature, specific EM strains added to the matrix (MSW and FW) have accelerated the process as well. Nakasaki and Hirai (2017), used the acid-consuming yeast *Pichia kudriavzevii* RB1 as inoculum for FW composting, which showed elimination of the lag phase and stimulation of microbiota; however, the quality of the final compost was unaffected. During FW composting, the role of lactic acid bacteria, essentially *Pediococcus* and *Weissella* has been elucidated well. It has been found that these bacteria accelerated the composting process, producing lactic acid, thus activating indigenous microorganisms within composting (Tran et al., 2019). Composting of FW conducted with organic acid-degrading mesophilic yeast inoculates, showed thorough degradation of the organic matter due to significant increase in number of certain groups of *Bacillus's* (Nakasaki et al., 2019). A study by Voběrková et al. (2017), where MSW was inoculated with consortium of white-rot fungi *Fomes fomentarius, Phanerochaete chrysosporium* and *Trametes versicolor*, assessed positive effect of EM inoculation on the enzymatic activities and compost quality. Analogous study on MSW composting with Bacillus isolates (*B. subtilis, B. tequilensis, B. venezuelans* and *B. amyloliquefaciens*), resulted in reduction of composting time and produced finest quality compost. Likewise, Ding et al. (2016) avoided acidification during the initial stage of waste composting by an inoculated bacterial consortium (anti-acidification) including *bacillus, lactobacillus, pseudomonas*, and others. This strategy achieved a superior quality compost with a higher humic acid content compared to control treatment. Relatively similar results were obtained in a waste composting set up, where an oil degrading thermophilic bacterial consortium was added to the treatments (Awasthi et al., 2017). It was noted that in all these experiments the characteristics of the raw material or substrate was considered for the respective inoculum to yield excellent results.

Despite cited literature, there were some reports that EM addition, single handedly does not ensure a quality compost, instead dependent on the compatibility of the microorganisms with the composted feedstock.

## 6 Compost maturity

The quality of compost vitally substantiates the overall productivity within a waste degradation process. At a closer look, “maturity” and “stability” are two important parameters that proficiently assessed the compost quality. Basically, the term ‘maturity’ validated the suitability of compost for agricultural purposes, subjected to its biological and chemical effects while, ‘stability’ was related indirectly to the biological activity, judging proper humidification of the organic fractions. However, very less information is comprehended about the universally accepted parameters to determine compost quality (Cofie et al., 2016). Certain parameters such as, changes in the physio-chemical properties (Santos et al., 2016), drop in compost temperature, C: N ratio, enzymatic activity, microbial activities and biomass (Tiquia et al., 2002), calorimetric and spectroscopic analysis of compost (De Oliveira et al., 2002), oxygen consumption (Tiquia et al., 2005), degree of self-heating capacity, phytotoxicity assays (Tiquia and Tam, 1998) germination tests (Zucconi et al., 1985), and cation exchange capacity are included to elucidate the quality of a compost.

Compost quality can induce fertility benefits (physical, chemical and biological), to prevent immobilization of nutrients and suppression of diseases in soil (Kutsanedzie et al., 2015). Although these indices are not very dependable, can help in providing rough idea about the same. The manifested physical variations in colour and odour of a substrate like, normally with time compost darkens (brown to dark brown) and odours becomes pleasant, transforming from offensive to ammonia-like and then finally earthy are relevant (Latifah et al., 2015). In addition, evaluation of the biological (microbial) stability can be monitored through metabolic activities, biomass and microbial count and quantity of biodegraded compounds (Barrena et al., 2014).

Chemical analysis for a compost included a detailed characterization of the composted wastes including pH, temperature, C/N ratio, moisture, organic matrix, and porosity level. Among these, temperature monitoring is the most simple and fast method to assess compost quality (Tiquia et al., 1996). A subsequent decrease in pile temperature at the end of composting, correlates well with other characteristics of a compost, used to evaluate stability or maturity in a compost. The C/N ratio is another key parameter to induce compost maturity within composting and co-composting processes (Golueke, 1991). The water soluble organic-C/total organic-N with a proposed value of <0.70, is considered suitable as per stability elucidation index.

Along with temperature and C/N, respirometric techniques (CO_2_ production) and plant bioassays also justified the compost maturity (Komilis and Kletsas, 2012). The respirometry activities, when deduced at a fixed temperature range (35-37 °C) are good indicators (evaluated by elemental analysis) to assess the metabolic potential of a compost (Maulini-Duran et al., 2014). An unstable compost demanded a higher O_2_ consumption, owing to the degradative capacity of biodegradable compounds in the feedstock. Soares et al. (2013), conducted respirometry (oxygen uptake rate) and bioassay tests during thermophilic composting of biowastes on *Lepidium sativum* seeds, while studying the lignin degradation within the water extracts as well. The results revealed that phytotoxicity and the period of compost maturation strongly influenced the mineralization process. Mokhtari et al. (2011), carried out an in-vessel MSW composting experiment and observed a decreased temperature, Specific Oxygen Uptake Rate (SOUR) (less than 2 mg O_2_/g) and nitrate nitrogen ratio with an acidic pH during the last stage of composting process. Similarly, compost stability of municipal sewage sludge evaluated through calculated oxygen uptake rate, showed an inverse relation with the basal respiration rate (Sciubba et al., 2015).

## 7 EM modified compost quality

The degree of humification is governed by structural chemistry of the fostered humic substances (HS), consisting of humic acids (HA) and fulvic acids (FA). While referring stability, several humification parameters, like humification index, humification and inclination in HA values due to oxygen depletion (Tiquia, 2005; Ko et al., 2008), were reported as good indicators of the compost stability. In addition, a lower percentage of water- and NaOH-extractable fulvic acids (FA), positively correlated compost maturity with the CO_2_ evolution (El Fels et al., 2014).

Few studies successfully assessed the impact of EM inoculation on the humification of lignocellulosic and cellulosic waste, and none assessed any negative effects on compost. In this context, bio-degradation of organic matter and lignocellulosic waste (dairy manure-sugarcane leaves co-composting) with optimized inoculation strategy, resulted in enhanced mineralization of organic carbon and accelerated lignocellulose degradation, achieving a good humification in waste (Xu et al., 2019). Another research put forward by Manu et al. (2017), produced ample quantities of humic and fulvic acids by inoculation of a commercial inoculum (yeast, phototrophic bacteria and lactic acid bacteria) to waste. A reduction in process time, enhanced rate of lignocellulose degradation, and improved compost quality were reported. Hachicha et al. (2012), observed similar results with fungus*, Trametes versicolor* as an inoculate to the lignocellulosic waste. An investigation on MSW composting by Rastogi et al. (2019a), stated the effect of cellulolytic bacterial additives on humic characteristics of waste. The study revealed an early maturity in the inoculated compost, owing to a good humification degree. Another study involving MSW composting, determined the effect of bacterial and fungal community dynamics on the humification of water extractable organic matter. The significant statistical relationship between humification and microbial dynamics of these special bacterial and fungal species at different composting stages, could be utilized successfully to monitor the process (Zhao et al., 2016; Zhao et al., 2017b). The correlation coefficients (r) between maturity and stability parameters were suggestive of multiple parameters (organic matter loss, C:N ratio, HA:FA ratio, HI, and NO_3_-N) to assess the maturity of different composts (Kutsanedzie et al., 2015).

Despite all the accounted benefits of compost, immature or low-quality compost can adversely affect the plant and soil environment (Fernandez-Hernandez et al., 2014). An immature and unstable compost can cause various problems like self-heating, a potent threat when heating occurred in a large heap due to uncontrolled microbial activity (Cerda et al., 2018). The application of an unstable and immature compost to soil can hinder nitrogen fixation, release toxic substances and restrict plant growth, competing for oxygen in the rhizosphere (Onwosi et al., 2017). In addition, they can cause phytotoxic effects and harm the seed germination, supress root growth, and hinder plant development. Some other major issues such as fires (leading to expansion and breaking), foul smell, diseases (vector attraction) and undesirable products (due to anaerobic conditions) are potentially toxic effects. Therefore, excellent results for small- and large-scale SW composting, can be achieved by confirming compost maturity through widely used stability indices (Maulini-Duran et al., 2014; Van Fan et al., 2016; Colón et al., 2017 and Van Fan et al., 2018a, Van Fan et al., 2018b).

## 8 Conclusion

A lot of information regarding the waste management technologies to tackle enormous waste quantities generated in India's urban areas is known. However, looking at the environmental implications of these methods, biological composting with an effective microbial culture (general or waste specific) seems an appropriate economic and ecofriendly way-out. An EM inoculated compost attains better compost quality and maturity in less process time period. These EM's can be isolated from variety of conventional sources such as soil, waste material or leachate and applied to the process at different stages (initial, mid or last). It is also suggested that governments play an active role in addressing the issues related to waste collection and segregation to implement a centralized SWMS. Compost quality is essentially computed to confirm non-toxicity of a compost towards plant growth. At last, excellent results for small- and large-scale SW composting, can be obtained by EM addition and compost maturity affirmation.

## Declarations

### Author contribution statement

All authors listed have significantly contributed to the development and the writing of this article.

### Funding statement

This work was supported by the University research scholarship scheme of Maharshi Dayanand University, Rohtak (Haryana), India.

### Competing interest statement

The authors declare no conflict of interest.

### Additional information

No additional information is available for this paper.
